# The Syringe Pump Gas Distribution (SPGD) system: a simple and low-cost method for simulating NH_3_/^15^NH_3_ deposition

**DOI:** 10.3389/fpls.2025.1460035

**Published:** 2025-03-28

**Authors:** Chunze Wu, Xing Wei, Chenghang Zhang, Saima Khan

**Affiliations:** ^1^ School of Forestry, Northeast Forestry University, Harbin, Heilongjiang, China; ^2^ Key Laboratory of Sustainable Forest Ecosystem Management-Ministry of Education, Northeast Forestry University, Harbin, China

**Keywords:** ammonia, chamber, dry deposition, forest ecosystem, isotope labeling, nitrogen deposition

## Abstract

Ammonia (NH_3_) in the atmosphere plays a crucial role in the global nitrogen cycle. Elevated NH_3_ deposition can result in various detrimental ecological and environmental consequences. Traditionally, researchers have employed methods such as static fumigation, dynamic fumigation using high-precision mass flow meters or standard gas cylinders, and free air enrichment to investigate vegetation responses to NH_3_ deposition. However, these approaches may suffer from inaccuracies, high costs, or technical complexity. In order to address this issue, we developed the Syringe Pump Gas Distribution (SPGD) system, a cost-effective new method for simulating NH_3_/^15^NH_3_ deposition. This system allows for precise and stable mixing of NH_3_/^15^NH_3_ stored in a syringe with air using a microinjection pump. The resulting mixture is then utilized to simulate NH_3_/^15^NH_3_ deposition. With just one 20 ml syringe, a single SPGD system can simulate NH_3_ deposition flux ranging from 0 to 31.74 mg N m^-2^ d^-1^ (equivalent to 0 - 116 kg N ha^-1^ yr^-1^) over an area of 0.36 m^2^. The SPGD system demonstrated reliability and stability during a 21-day simulated deposition test on potted *Populus cathayana* under greenhouse conditions (including simulated rainfall). It exhibited adequate adjustment resolution to generate environments with varying NH_3_ concentrations, corresponding to different NH_3_ deposition fluxes. The test findings indicated a positive correlation between the δ_15_N levels in *P. cathayana* leaves and the NH_3_ deposition flux increase. The cost, complexity, and risk associated with simulating NH_3_ deposition can be significantly decreased by utilizing the SPGD system. The SPGD system is modular (gas supply unit and NH_3_ supply unit) and can be adapted to different research needs, including for simulating the deposition of NO_2_, SO_2_ or mixtures. Adopting this system, researchers can safely and efficiently simulate NH_3_ deposition or perform ^15^NH_3_ labeling, thereby advancing the understanding of physiological and ecological processes associated with plants and even forest ecosystems under gaseous deposition.

## 1 Introduction

Ammonia (NH_3_) is an important component of the global nitrogen cycle, which plays an important role in all ecosystems. Since the mid-20th century, emissions of NH_3_ to the atmosphere have continued to increase on a global scale due to increased intensive agricultural activities and animal feedlot operations (Warner et al., 2017; Li et al., 2016). The increased deposition resulting from these emissions has led to various detrimental environmental impacts, particularly affecting forest ecosystems. These impacts include eutrophication, acidification, and direct toxicity to ecosystems, raising concerns globally (Van der Eerden et al., 1992; Aber et al., 1998; Lu et al., 2014).

Atmospheric NH_3_ can be absorbed, assimilated and utilized by the leaves of higher plants, leading to changes in plant physiology and function. Since the 1980s, scientists have worked to understand the response of different vegetation to simulated atmospheric NH_3_ deposition. This involves creating environments with constant NH_3_ concentrations or specific NH_3_ supplies to evaluate the potential impact of projected increases in real atmospheric NH_3_ deposition on forest ecosystem structure and function. However, our understanding of vegetation responses to deposited NH_3_ remains limited, partly due to the scarcity of cost-effective, simple, and reliable methods for simulating NH_3_ deposition on a field or laboratory scale.

Supplying a certain amount of pure NH_3_ gas in a chamber can simulate NH_3_ deposition to some extent, but the simulation time is limited and the NH_3_ concentration is uncontrollable (Paoli et al., 2015; Huang et al., 2020). One approach to overcome these limitations is to continuously provide NH_3_ to greenhouses, indoor controlled environment chambers or open top field chambers (OTCs) using dynamic gas distribution methods (i.e., a continuous mixture of a source gas of known concentration and a purified diluent gas in a ratio to obtain a continuous rationed supply of a standard gas). However, the relative rate of supply of NH_3_ in dynamic gas distribution methods tends to be low due to the natural low NH_3_ concentrations in nitrogen deposition (Warner et al., 2017; Liu et al., 2020; Tanner et al., 2022), leading to complexity and increased costs. For example, in previous studies, people used configured standard gases with a low NH_3_ concentration as the NH_3_ source to expand the supply rate of NH_3_ (e.g., a cylinder containing with a calibrated gas mixture of 0.1% NH_3_ in N_2_) (Wu et al., 2023), increased the supply rate of the dilution gas to expand the supply rate of NH_3_ (Adrizal et al., 2006), or used high-precision NH_3_ mass flowmeters to meet the precise control of the NH_3_ supply (Castro et al., 2006; Chen et al., 2020). An alternative approach is similar to FACE (free-air carbon dioxide enrichment) experiment (Sheppard et al., 2011; Deshpande et al., 2024). Simply put, NH_3_ is supplied from a cylinder of pure compressed liquid NH_3_ and diluted with ambient air. Although this approach has the advantage of being closer to the real environment and simultaneous studies of many biological scales and processes, scaling up this approach to the field is challenging due to its high cost.

In addition, NH_3_ enriched in a stable isotope of nitrogen (^15^NH_3_) is an accurate and convincing tool for quantifying the absorption and distribution of NH_3_ by plants. In previous studies, the simulation of ^15^NH_3_ deposition was mostly achieved by injecting a certain amount of pure ^15^NH_3_ or its reaction substrate into the chamber (Tomaszewski and Sievering, 2007; Bazot et al., 2008; Silva et al., 2015). A continuous and controllable supply of ^15^NH_3_ will undoubtedly increase the difficulty and cost of simulation. Jones et al. (2008) used peristaltic pumps to continuously mix NaOH and (^15^NH_4_)_2_SO_4_ solution, and then used the ^15^NH_3_ generated by the reaction of the mixed solution to simulate the ^15^NH_3_ deposition. Although this ingenious approach achieves the above conditions, the generation of ^15^NH_3_ in this approach is limited by the flow rate of the air used for mixing.

Overall, it is necessary to provide a new simple, low-cost and safe solution for the simulation of NH_3_/^15^NH_3_ deposition in greenhouses, indoor controlled environment chambers or OTCs. In this study, we devised and tested the Syringe Pump Gas Distribution (SPGD) system for simulating NH_3_/^15^NH_3_ deposition. This system allows for precise mixing of high-purity NH_3_/^15^NH_3_ with air using a microinjection pump, enabling accurate simulation of NH_3_/^15^NH_3_ deposition. We verified the feasibility of the SPGD system by performing NH_3_/^15^NH_3_ deposition treatment on *Populus cathayana* seedlings within the chamber. We describe in detail the components involved in SPGD system and its simpler and safer simulation process of NH_3_/^15^NH_3_ deposition.

## 2 Materials and methods

### 2.1 NH_3_/^15^NH_3_ generation and collection in the laboratory

We added well-mixed NH_4_Cl/^15^NH_4_Cl and Ca(OH)_2_ solids in a test tube and allowed them to react under heating conditions. The generated NH_3_/^15^NH_3_ was dried by soda lime and collected in a Teflon- aluminum foil gas sampling bag (see **Supplementary Figure S1** for a schematic diagram of the reaction apparatus). The concentration of NH_3_/^15^NH_3_ in the syringes, determined through Water Absorption, was found to exceed 90% (**Supplementary Figure S2**).

### 2.2 Syringe Pump Gas Distribution system: components, installation and operation

**Figure 1:**
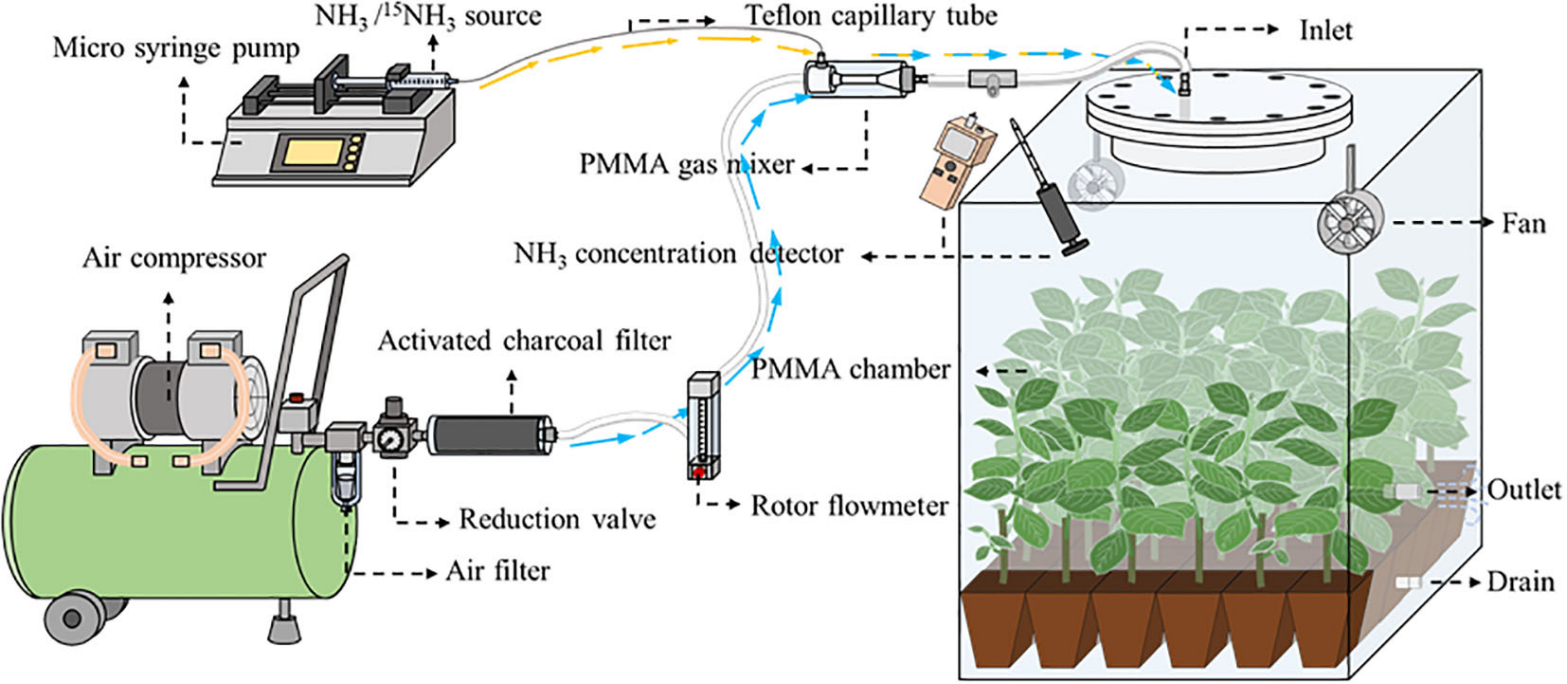
A schematic diagram showing the Syringe Pump Gas Distribution (SPGD) system. Within the chamber are *Populus cathayana* potted seedlings. PMMA, polymethyl methacrylate. The yellow arrow and the blue arrow represent the flow direction of ammonia and air in the tube respectively.

**Figure 2:**
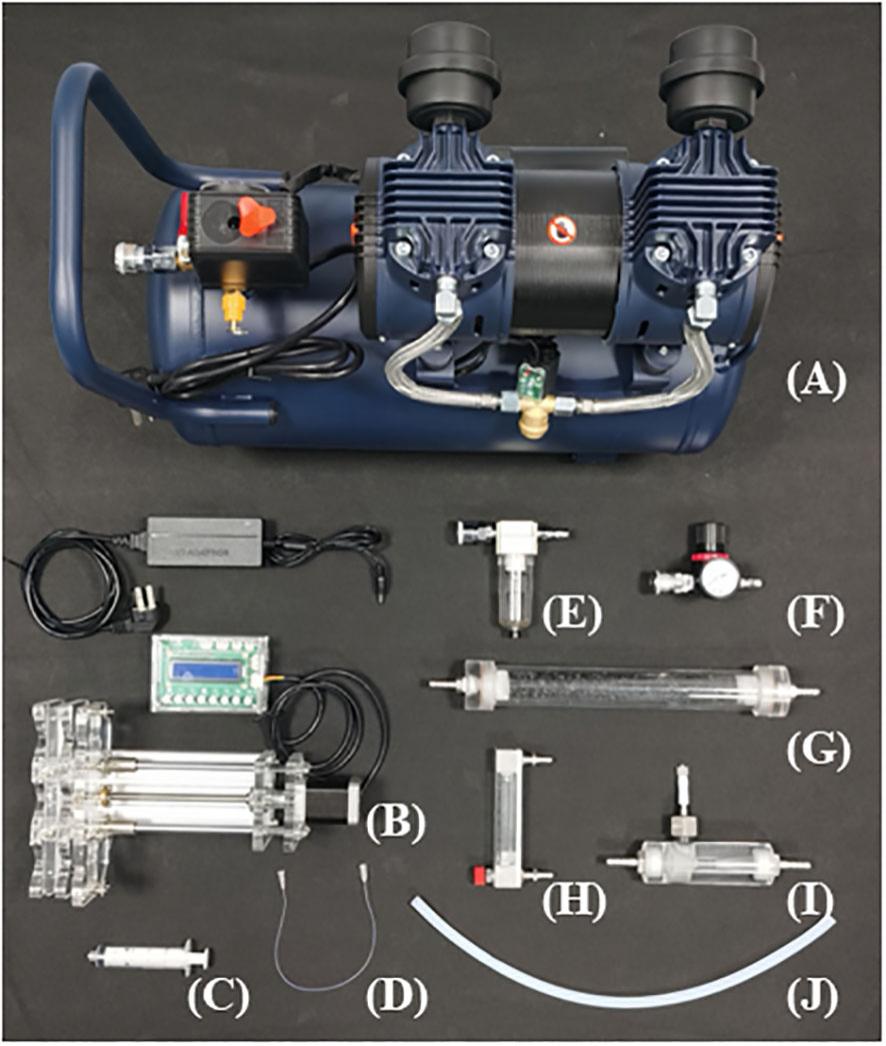
Basic components of the Syringe Pump Gas Distribution (SPGD) system: **(A)** Air compressor; **(B)** Microinjection pump; **(C)** Syringe; **(D)** Capillary tube; **(E)** Water separator; **(F)** Reduction valve; **(G)** Activated charcoal filter; **(H)** Rotor flowmeter; **(I)** Venturi gas mixer; **(J)** Gas tube.

The SPGD system is mainly composed of air supply unit, NH_3_ supply unit and chamber. Briefly, the ambient air provided by the air compressor is continuously mixed with the NH_3_ provided by the microinjection pump, and the mixed gas is used to simulate NH_3_ deposition in the chamber (**Figure 1**). In the SPGD system, except for the chamber (see below), all components are common pneumatic or laboratory supplies. The total cost of a single system is approximately $249. The following are the installation instructions for the components in each unit and the operating instructions for the SPGD system (see **Figure 2** for basic components and **Supplementary Table S1** for bill and specifications of components).

**Figure 3:**
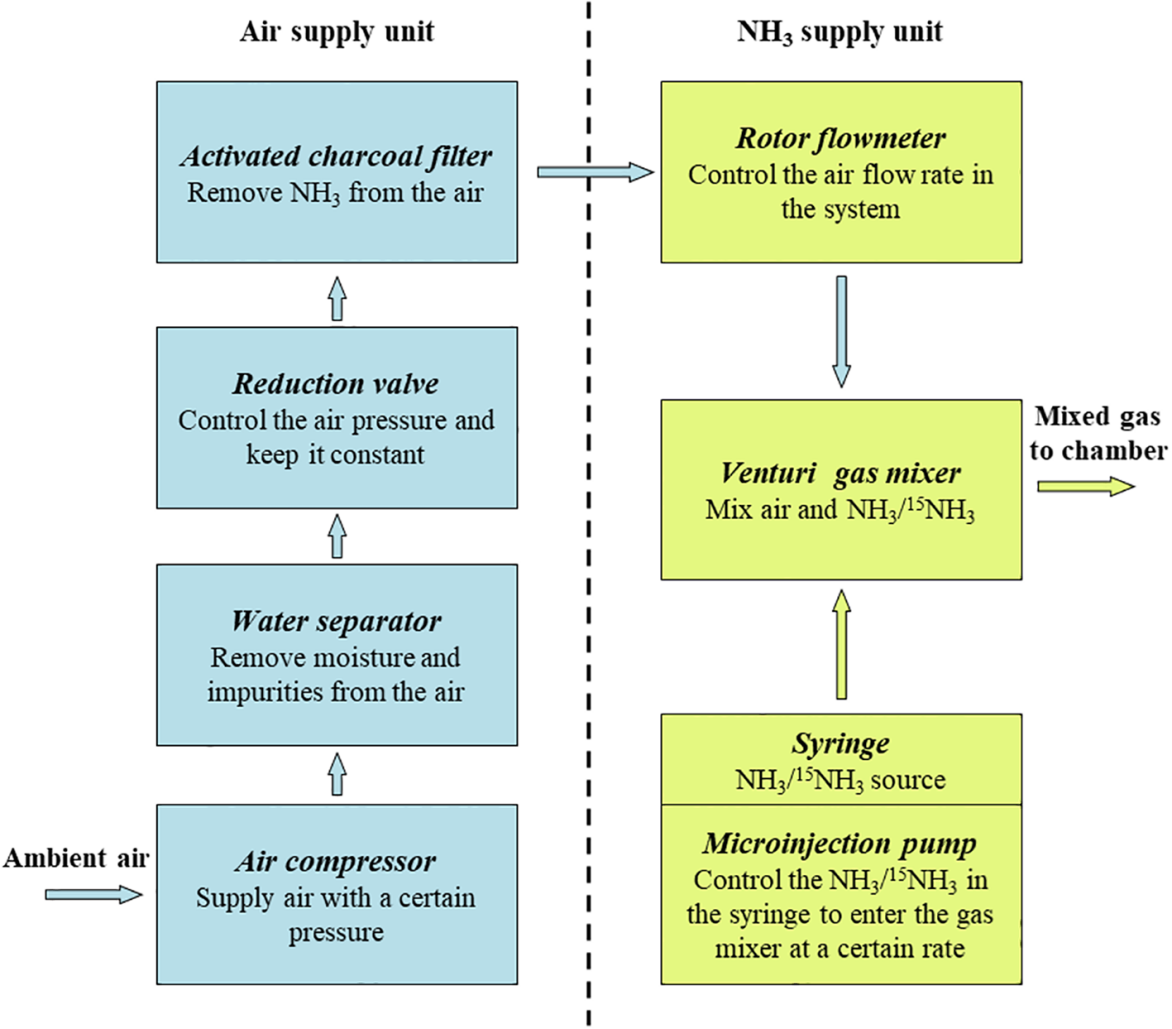
Principle of operation of the Syringe Pump Gas Distribution (SPGD) system and functions of the main components. The yellow arrow and the blue arrow represent the flow direction of ammonia and air in the system respectively.


1. Air supply unit: The air supply unit is mainly composed of an air compressor, a water separator, a reduction valve and an activated charcoal filter. The ambient air compressed by the air compressor is pumped into Venturi gas mixer after passing through the water separator, reduction valve, activated charcoal filter and rotor flowmeter in turn (**Figure 1**). The flow rate of the output air from the air compressor is controlled by adjusting the rotor flowmeter and is kept uniform by the reduction valve (**Figure 3**). Pneumatic quick connectors, pagoda connectors or polyethylene tubes are used to connect the components. The selection of specifications for each component of the air supply unit depends on the air exchange rate (i.e. air flow rate) in the deposition. In this study, for an air flow rate of 30 L min^-1^, we chose an air compressor with a power of 1580 watts; a pressure-reducing valve, a moisture filter, and an activated charcoal filter with inlet and outlet thread sizes of 13.2 mm (1/4-inch threads); and 8*10 mm polyethylene tubes. The reason we choose the Venturi gas mixer is to avoid the influence of the high pressure of the air supply unit on the NH_3_ supply unit. See **Supplementary Figures S3**-**S6** for the connection guide of each component.
2. NH_3_ supply unit: The NH_3_ supply unit is mainly composed of a microinjection pump and a syringe. The prepared NH_3_/^15^NH_3_ in the syringe is continuously pumped into the gas mixer through a microinjection pump. The operating characteristics of the microinjection pump (i.e., uniform motion throughout, smooth and pulsation-free) allow for precise control of the NH_3_ flow rate at its output (**Figure 3**). Microinjection pump propulsion linear velocity adjustment resolution and the minimum value of linear velocity range should not be greater than 0.01 mm min^-1^. The syringe and the gas mixer are equipped with Luer connectors, and they are connected with 0.2*0.4 mm stainless steel needles and 0.3*0.6 mm polytetrafluoroethylene (PTFE) capillary tubes. The gas mixer and the chamber are equipped with a pagoda connector, and they are connected with 8*10 mm PTFE tubes. The unit is placed in a modified storage box, which is designed to be easy to handle as well as dustproof (**Supplementary Figure S7**).
3. The open-top chamber in this study was made of polymethyl methacrylate (PMMA), with flange links at the openings. The air inlet is located at the top of the flange cover and the air outlet and drain are located at the bottom of the chamber. Two fans are installed in the chamber to reduce the difference in NH_3_ concentration over horizontal space.
4. Simulation of deposition: Here we outline the instructions for operating the SPGD system. After powering on all equipment, start by turning on the air pump switch to inflate the air pump. Next, extract the NH_3_ in the gas collection bag using a syringe and then install the syringe on the syringe pump. Finally, operate the syringe pump and air pump while adjusting the flow rates of NH_3_ and air. When running the SPGD system, it is important to minimize the pressure difference between the reduction valve, activated charcoal filter, and the rotor flowmeter in order to prevent the formation of condensation water inside the activated charcoal filter and rotor flowmeter. In addition, the air tank of the air compressor should be drained and the filter element in the water separator should be replaced regularly. In this study, we regulated the NH_3_ concentration in the gas mixture by adjusting the syringe pump propulsion linear velocity, and assumed that the NH_3_ in the syringe pump was pure when calculating NH_3_ concentration. The NH_3_ concentration was calculated as follows:

$$\rho_{N}=(\frac{S_{S}\times Q_{l}\times 14}{V_{m}\times Q_{L}})\times 1000$$

Where ρ_N_ is the concentration of NH_3_ (μg N m^-3^), *S*_S_ is the cross-sectional area of the syringe inner diameter (mm^2^), *Q*_l_ is the linear velocity of the injection pump (mm min^-1^), 14 is the molar mass of nitrogen atom (g mol^-1^), *V*_m_ is the molar volume of gas (L mol^-1^), *Q*_L_ is the total gas flow rate in the system (L min^-1^). For example, at a temperature of 25°C (with a molar volume of 24.5 L mol^-1^), and a total gas flow rate of 60 L min^-1^, utilizing a syringe with an inner diameter of 19 mm and a syringe pump with a linear velocity resolution of 0.01 mm min^-1^, the calculated theoretical minimum resolution for adjusting NH_3_ concentration is approximately 27 μg N m^-3^.

5. Maintenance of the SPGD system: In the SPGD system, the rotor flowmeter, microinjection pump and reduction valve are key components for supplying a stable and controllable concentration of NH_3_ (**Figure 3**). Therefore, regular calibration of the rotor flowmeter, the microinjector pump and the reduction valve is essential to ensure the accuracy and stability of the SPGD system in long-term use. In this study, for the rotor flowmeter, the error was calculated by comparing the time required for the gas output from the flowmeter to fill a gas collection bag of known volume with the preset value. For the microinjection pump, the error was calculated by comparing the propulsion travel of the microinjection pump per unit time with the preset value. The error of syringe pumps and flow meters was controlled within ± 5%, beyond which the equipment was repaired or replaced. In a normal operating reduction valve - rotor flowmeter system, the gas flow rate indicated by the flowmeter was stable and free of fluctuations. Therefore, the calibration of the reduction valve was determined by observing the reading of the rotor flowmeter. In addition, other components in the SPGD system were checked regularly to ensure the functional integrity of the SPGD system. After calibration or replacement of components, the NH_3_ concentration output by the system was tested and compared with the theoretical value to verify the accuracy of the maintained SPGD system.

## 3 Test in the greenhouse and results

**Figure 4:**
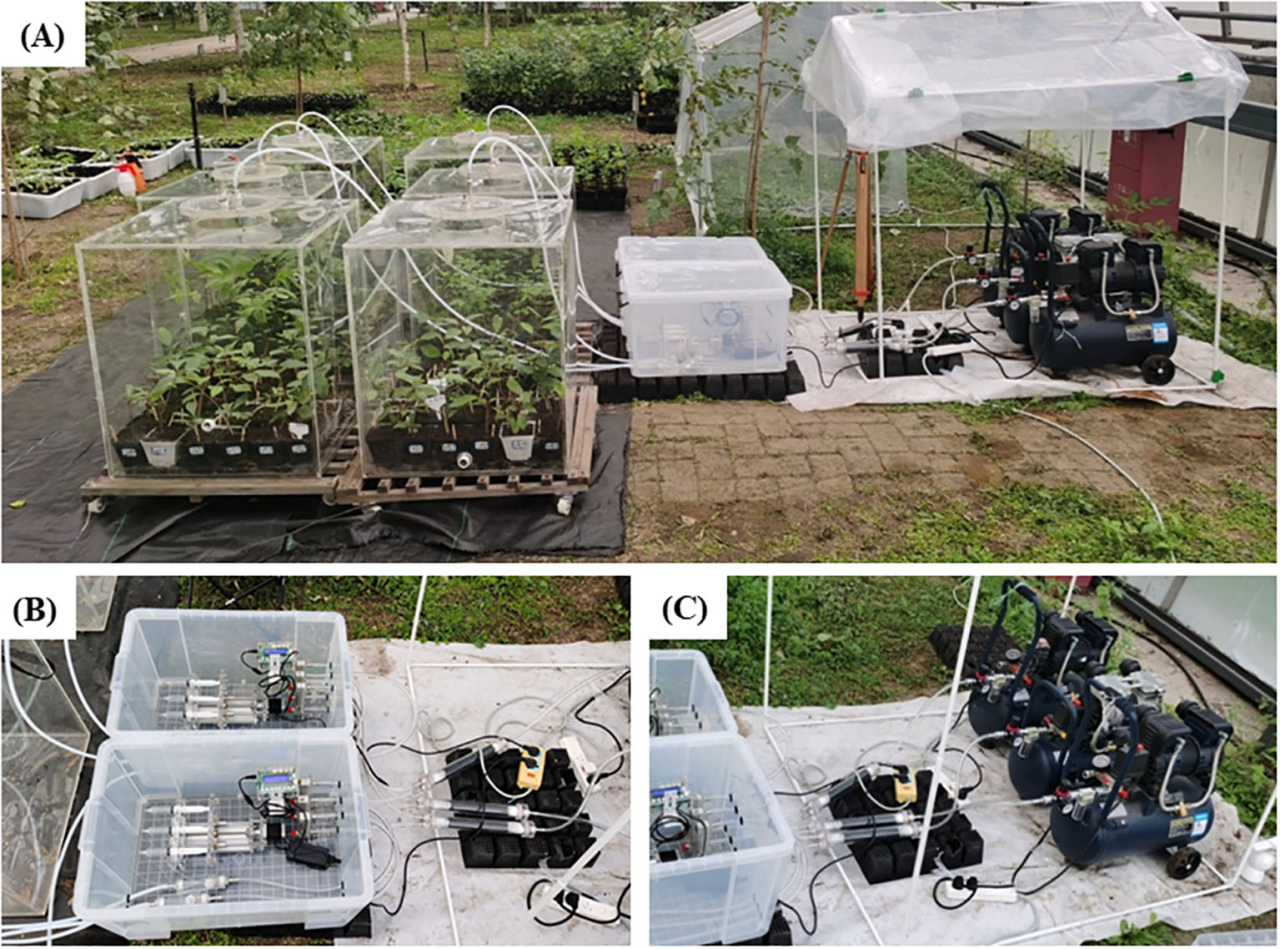
The Syringe Pump Gas Distribution (SPGD) system set up in the greenhouse: **(A)** SPGD system as a whole; **(B)** NH_3_ supply unit; **(C)** Gas supply unit.


From August 1 to August 21, 2023, we conducted experiments using the SPGD system to test 1-year-old *P. cathayana* seedlings in a greenhouse (with simulated rainfall) (**Figure 4**). The SPGD system was operated from 6:00 to 18:00 each day during the test. We established three pre-determined NH_3_ concentration treatments: Control (0 μg N m^-3^, no NH_3_ applied), LN (216 μg N m^-3^), and HN (432 μg N m^-3^). Each concentration had two systems, totaling six chambers. The air flow rate for each system was 30 L min^-1^, with 36 seedlings in each chamber. On August 21, 2023, we replaced NH_3_ with ^15^NH_3_. During the test, the temperature in the greenhouse was 23-34 °C and the relative humidity was 60-100% (90%-100% during simulated rainfall). During the operation of the SPGD system, the maximum light intensity in the chamber was 23,600 lux, the average temperature was 28.5 °C, and the relative humidity was 40-60%. On the first day of the test, we measured the NH_3_ concentrations at the inlet and outlet of the chamber every 2 h using a pump suction NH_3_ detector (JA908 NH_3_; Yongqi Co. Ltd, GuangDong, China; Accuracy, ≤ ± 3% F.S; Detection limit, 20 ppb) and NH_3_ detection tube (3CG Ammonia; GASTEC, Tokyo, Japan; Detection limit, 0.02 mg m^-3^), and collected ambient temperature and relative humidity every 1h at a distance of 1.5 m above the floor and 5 m from the SPGD system (**Supplementary Figure S8**). Before the test, the correction factor for NH_3_ adsorption on the chamber inner wall was determined by running the SPGD system without plant-soil material, and the air flow rate was set to 30 L min^-1^. To calculate the correction factor, the factor of NH_3_ adsorption on the inner wall of the chamber was calculated:

$$\rho_{C}=\rho_{In}-\rho_{Out(empty)}$$

Where ρ_C_ is the factor of NH_3_ adsorption on the inner wall of the chamber (μg N m^-3^), ρ_In_ is the measured NH_3_ concentration at the inlet of the chamber (μg N m^-3^), ρ_Out(empty)_ is the measured NH_3_ concentration at the outlet of the chamber without plant-soil material (μg N m^-3^).

The correction factor was then obtained by calculating the difference between the total area of the chamber inner wall and the vertical projection area of the soil-plant material in the chamber after the start of the test:


$$\rho_{X}=\rho_{C}\times (1-\frac{S_{P}}{S_{C}})$$

Where ρ_X_ is the correction factor (μg N m^-3^), *S*_P_ is the vertical projection area of the plant-soil material (m^2^), the inner wall of the chamber covered by this area is considered to be free of NH_3_ adsorption. *S*_C_ is the total area of the chamber inner wall (m^2^).

After obtaining the correction factor (about 40 μg N m^-3^, see **Supplementary Table S2**, **Supplementary Figure S9** for detail parameter values), the NH_3_ deposition flux was calculated from the difference of measured NH_3_ concentration between the inlet and outlet of the chamber:


$$F_{N}=(\rho_{In}-\rho_{Out}-\rho_{X})\times \text{Q}\times \text{T}\times \frac{1}{S_{P}}\times 10^{-6}$$

Where F_N_ is the NH_3_ deposition flux (mg N m^-2^ d^-1^) and is defined as the NH_3_ retained by the plant-soil system in the chamber. ρ_Out_ is the measured NH_3_ concentration at the outlet, *Q* is the total gas flow rate (L min^-1^), which is consistent with the flow rate when calculating ρ_X_, *T* is the SPGD system operation time per day (min d^-1^).

After the SPGD system had been working for 40 min, we used the detector (connected a gooseneck tube) to detect the NH_3_ concentration at three horizontals in the chamber to determine the degree of mixing between NH_3_ and air: 5 cm (below the canopy), 35 cm (in the canopy), and 55 cm (above the canopy) from the soil surface in the chamber. The mean NH_3_ concentrations were collected at four points at each horizontal. Every 5 days, we measured the inlet and outlet concentrations at the beginning of the experiment using the pump suction NH_3_ detector. In addition, we tested the performance of the SPGD system in creating NH_3_ concentrations at air flow rates of 15-100 L min^-1^.

On the last day of testing, we collected poplar leaves for analysis of δ^15^N. All leaves of 3 poplar seedlings were harvested immediately after ^15^NH_3_ deposition, and placed in a freezer (0-4°C) and brought back to the laboratory. In the laboratory, the tissue was oven-dried at 65°C until constant weight for N isotope measurements. All samples were ground into fine powders using a ball mill (MM400; Retsch, Haan, Germany). The N concentration and N stable isotope (atomic percentage of ^15^N and δ^15^N) were determined using the isotope ratio mass spectrometer (MAT 252; Thermo Electron, Bremen, Germany). Analytical quality was checked by standards (glycine) with known isotope abundance. The international standard for N is atmospheric N_2_ with an R (^15^N/^14^N) value of 0.003676. Instrument precision was 0.15‰ for δ^15^N. NH_3_ concentration and leaf δ^15^N were assessed using one-way ANOVA, and least significant difference tests were conducted to compare means of these. All statistical analysis mentioned above was performed using the SPSS software v19.0 (SPSS Inc., Chicago, IL, USA).

**Figure 5:**
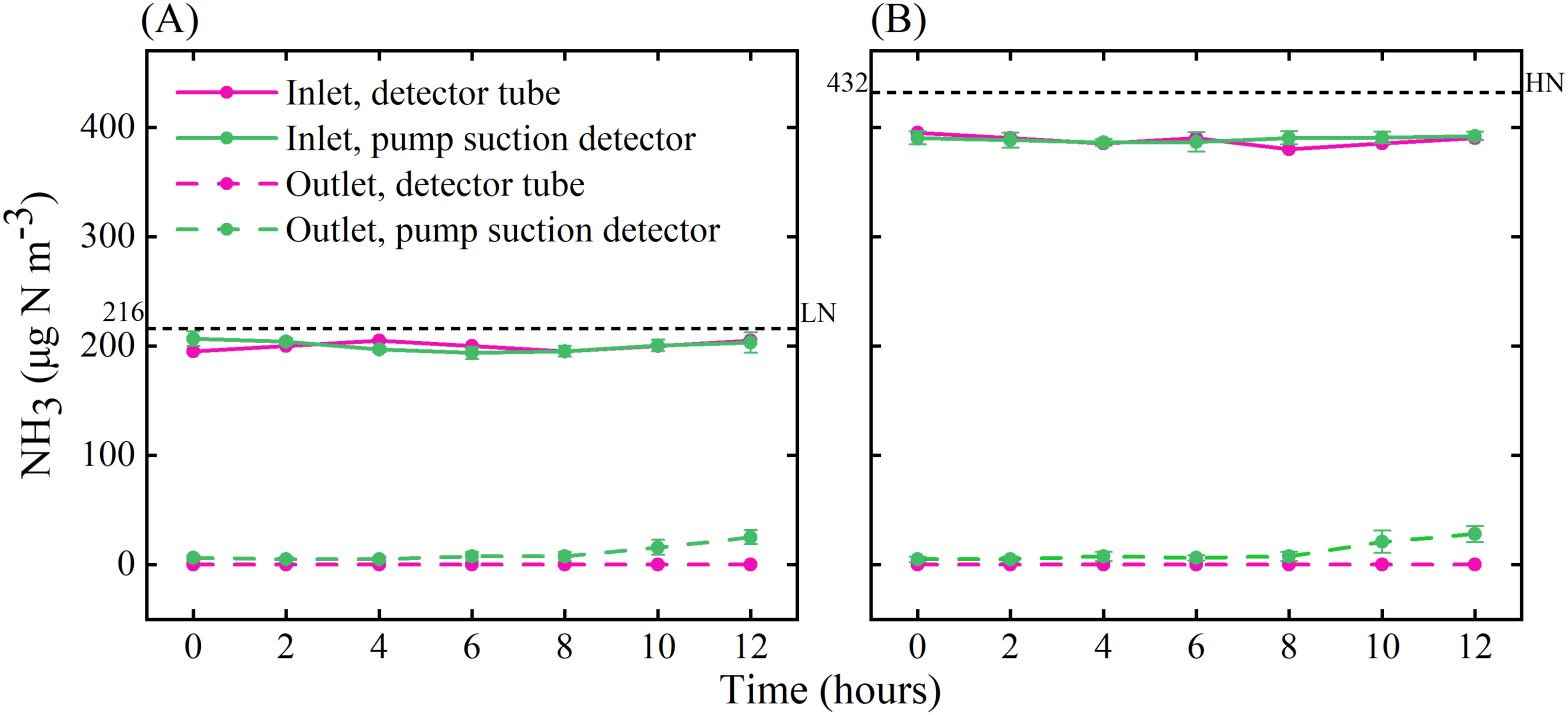
NH_3_ concentrations at the inlet and outlet of the chamber under LN **(A)** and HN **(B)** treatments. The black dotted lines indicate the theoretical value of NH_3_ concentration, the pink solid and dotted lines are the results from the pump suction detector, and the green solid and dotted lines are the results from the gas detector tube. Error bars indicate the standard error of the mean of NH_3_ concentration (*n* = 3).

**Figure 6:**
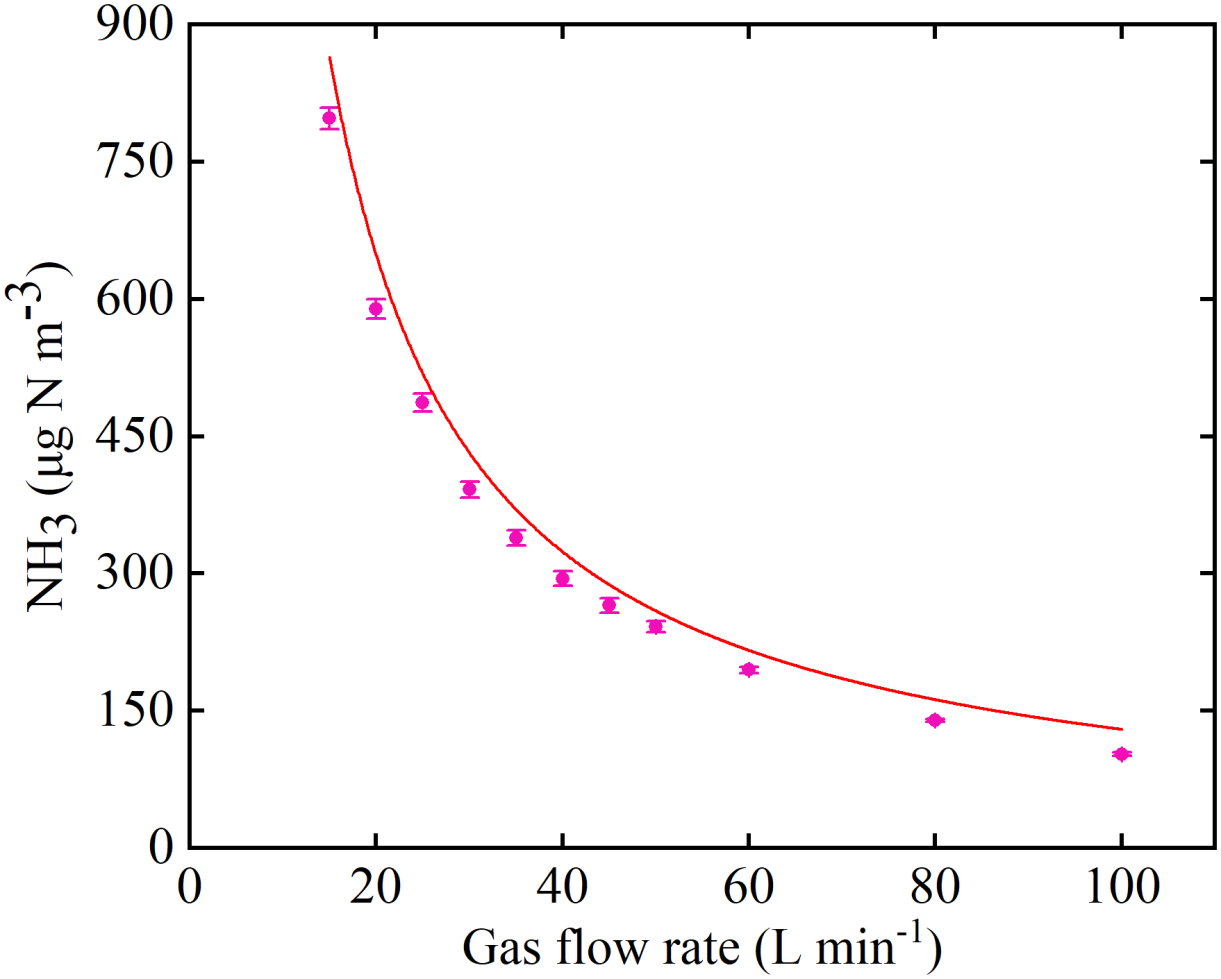
NH_3_ concentration output by the Syringe Pump Gas Distribution (SPGD) system at different gas flow rates. The red dashed line represents the theoretical expectation, based on the formula given in the study and assuming that the NH_3_ in the syringe is pure.

**Figure 7:**
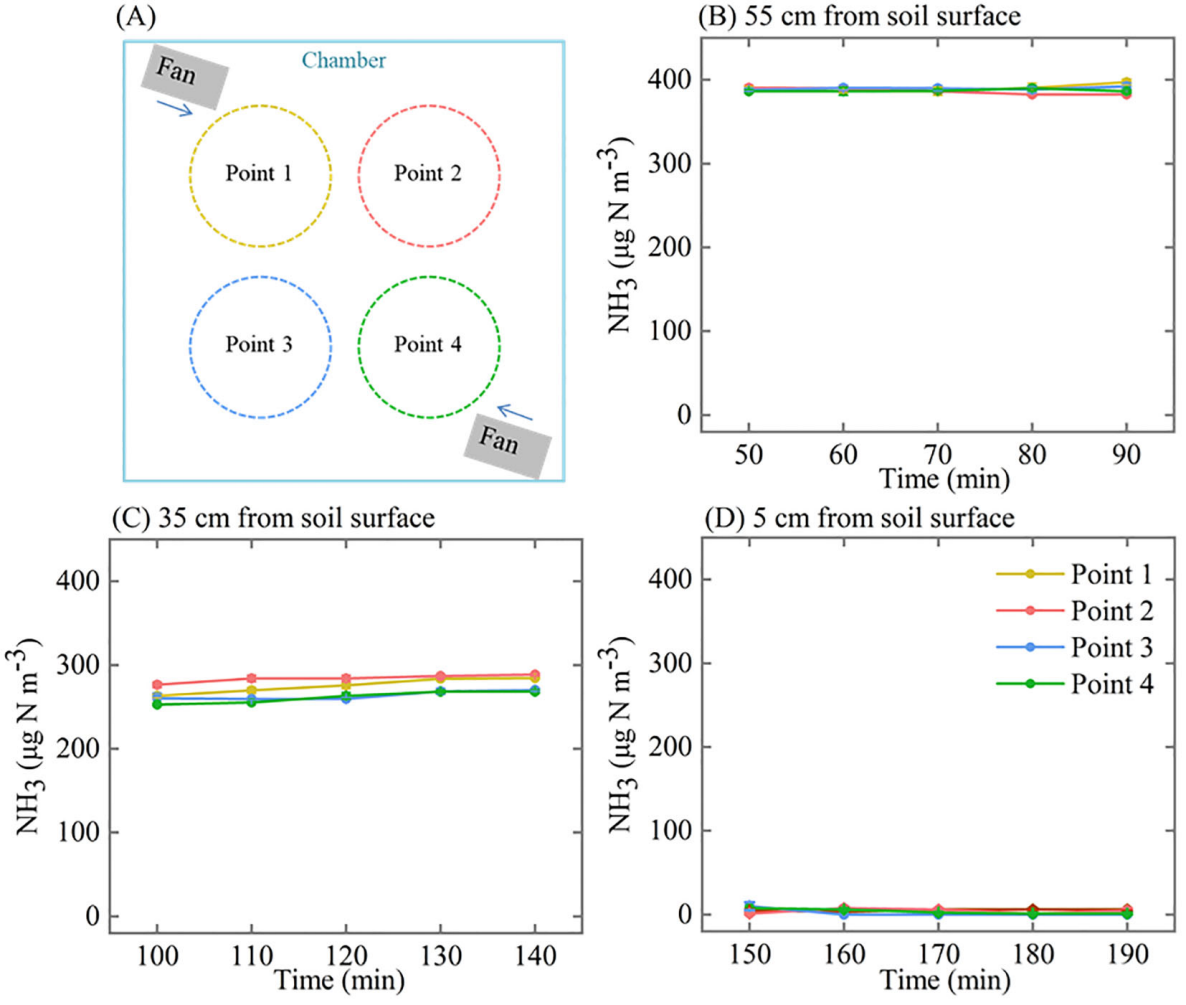
NH_3_ concentrations at three horizontals in the chamber: **(A)** location of measurement points per horizontal; **(B)** 55 cm from the soil surface, above the canopy; **(C)** 35 cm from the soil surface, in the canopy; **(D)** 5 cm from the soil surface, below the canopy. Error bars indicate the standard error of the mean of NH_3_ concentration (*n* = 3).

**Figure 8:**
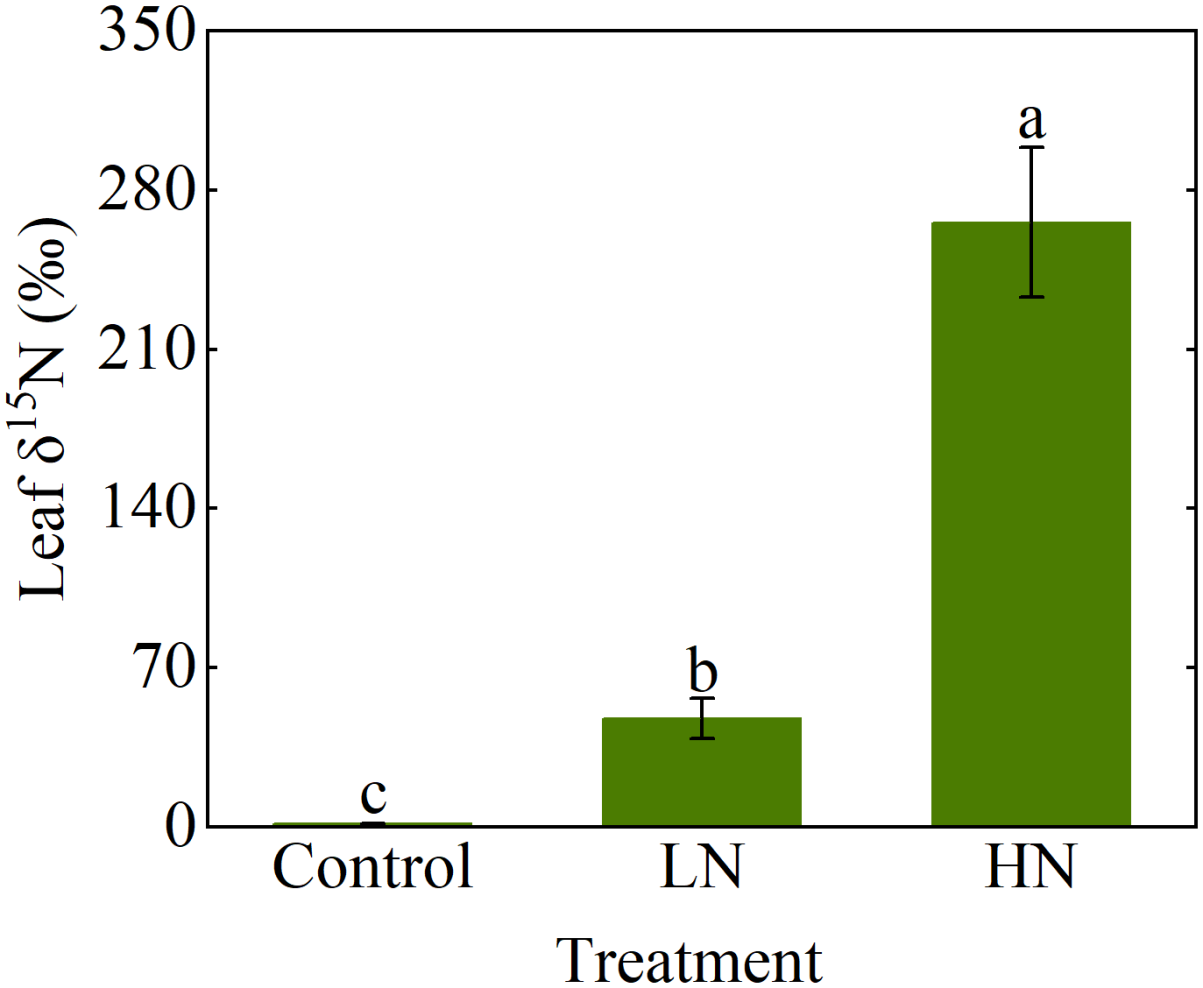
δ^15^N in *Populus cathayana* leaves affected by three ammonia (NH_3_) deposition treatments. NH_3_ deposition treatments included a control (0 μg N m^-3^, no NH_3_ applied), LN (216 μg N m^-3^), and HN (432 μg N m^-3^). Error bars indicate the standard error of the mean of δ^15^N (*n* = 3). Different letters above the error bar represent significant differences among three treatments (*P* < 0.05) according to the least significant difference test.

Measured NH_3_ concentrations at the inlet indicated that the system could be operated stably for at least 12 h during the day (**Figure 5**), and there was no significant difference in the NH_3_ concentration during the 21 days of operation (**Supplementary Figure S10**). When the microinjection pump propulsion linear velocity remains constant, the NH_3_ concentration output by the system decreases in a power function as the flow rate increases (**Figure 6**). The NH_3_ concentrations at the inlet and outlet were 0 μg N m^-3^ in the Control treatment (the presence of NH_3_ was not detected by either detector), but the NH_3_ concentrations in the LN and HN treatments were lower than the theoretical values of about 16 μg N m^-3^ and 42 μg N m^-3^, respectively (**Figure 5**). The NH_3_ concentration at the outlet in both the LN and HN treatments was less than 28 μg N m^-3^. Therefore, the NH_3_ deposition fluxes in the chamber were calculated to be about 9.61 mg N m^-2^ d^-1^ and 21.01 mg N m^-2^ d^-1^, equivalent to 35 kg N ha^-1^ yr^-1^ and 77 kg N ha^-1^ yr^-1^, respectively. In addition, the mean NH_3_ concentrations at each horizontal in the chamber had no significant difference between different measurement points or at different times (**Figure 7**). The isotopic results showed that δ^15^N of leaf tissue was significantly increased in LN and HN treatments compared to Control treatment, and showed an increasing trend with the increase of NH_3_ deposition flux (**Figure 8**).

## 4 Discussion

### 4.1 Comments on the SPGD system

During the 21-day, 12-hour-a-day test, the NH_3_ concentration output by the SPGD system was stable and was not affected by changes in temperature and humidity in the environment (**Figure 5**; **Supplementary Figure S8**). In comparison to methods utilizing standard gas cylinders or high-precision mass flow meters, the SPGD system offers a lower cost (including maintenance costs), increased safety profile, and is equally accurate for creating a certain concentration of NH_3_ (**Table 1**). This is attributed to the utilization of the low-cost and high-precision microinjection pump and the preparation of near-pure NH_3_/^15^NH_3_ in the laboratory, eliminating the need for hazardous high-pressure gas cylinders and reducing NH_3_/^15^NH_3_ waste. Compared to the cumbersome adjustment of the flow rate of the NH_3_ source when using gas cylinders, the microinjection pump has a simpler operating process (e.g., its pre-set flow rate and run time, etc.). One person can complete the simulated deposition of 6 systems in less than 12 min during the test. Compared with gas cylinders or peristaltic pumps, syringes and microinjection pumps are more convenient to install, so the SPGD system has greater convenience and higher applicability in the establishment of NH_3_ deposition simulation (**Table 1**). In this study, the actual NH_3_ concentration created by the SPGD system was 89.4% to 95.6% of the theoretical value obtained by calculation (**Figure 5**; **Supplementary Figure S2**), which is consistent with the actual NH_3_ concentration in the syringe (i.e. exceed 90%). Therefore, the assumption that the NH_3_ in the syringe is pure is not valid. The difference between the actual NH_3_ concentration and the assumed concentration during the calculation (i.e. pure) in the syringe caused the NH_3_ concentration created by the SPGD system to differ from the theoretical value. In fact, this also confirmed the excellent performance of the SPGD system in terms of air tightness. More standardized NH_3_ production operations and syringe use (e.g., multiple flushing the gas collection bag, syringes, and related pipes with prepared NH_3_ to reduce the residual of pre-existing air) may increase the concentration of NH_3_ in the syringe and reduce the difference between the created NH_3_ concentration and the theoretical value.

**Table 1:** Summary of simulation methods for NH_3_ deposition.

Simulation Methods	Accuracy	Cost	Convenience	Deposition area	Scalability	Safety	Use of gas cylinders
Free air NH_3_ enrichment	**+**	**+++**	**+**	**+++**	**+**	**++**	⚡
Based on mass flow meter	**+++**	**++**	**++**	**++**	**++**	**++**	⚡
Based on standard gas	**+++**	**++**	**++**	**++**	**++**	**++**	⚡
Simple gas addition	**++**	**+**	**+++**	**+**	**+**	**+++**	□
Based on peristaltic pump	**+++**	**+**	**++**	**++**	**++**	**+++**	□
SPGD system	**+++**	**+**	**+++**	**++**	**+++**	**+++**	□

+, Low; ++, Medium; +++, High; ⚡, Yes; □, No.

The disadvantage of the SPGD system is that its air supply unit inability to protect against dust and water. Although we used a rain shelter to block simulated precipitation in the greenhouse in this study (**Figure 4**), the use of the SPGD system under more complex conditions still faces challenges. For example, long-term rainfall may cause the electrical components of the air compressor to short-circuit, and the accumulation of dust may block the air inlet of the air compressor, etc. We recommend that the gas supply component of the system be placed in a relatively sealed space to accommodate its potential use in more complex conditions (such as in the field). Furthermore, while the closed chambers we used provide more precise control over deposition flux compared to free air enrichment and OTCs, they can experience excessively high internal temperatures (up to 50 °C) under full-light conditions in the field. Therefore, the customized chamber with controllable temperature is a potential solution to the limitation of high temperature. We found that the difference in NH_3_ concentration between the four points in the canopy at the same time was larger (although not statistically significant) compared to the other two horizontals (**Figure 7**). This may be due to different NH_3_ adsorption potentials resulting from differences in canopy structure. From a chamber design perspective, more fans or higher gas exchange rates within the chamber may help to reduce the difference in NH_3_ concentration over horizontal space (Jones et al., 2007).

### 4.2 Applicability of the SPGD system

The NH_3_ concentration output by the system in the gas flow rate range of 15-100 L min^-1^ was in line with theoretical expectations (**Figure 6**). Therefore, the SPGD system can meet the demand for different gas flow rates (i.e., gas exchange rates) in other studies. The excellent performance of the SPGD system under the environmental conditions of this study also shows that the system can be applied to most indoor or greenhouse research (**Figure 5**; **Supplementary Figure S8**). A single SPGD system has the capability to achieve a maximum deposition flux of 31.74 mg N m^-2^ d^-1^ (equivalent to 116 kg N ha^-1^ yr^-1^) over an area of 0.36 m^2^ by utilizing one 20 ml syringe per day. This makes it particularly suitable for research involving herbaceous plants, saplings, or branch materials. If a larger deposition area is needed, it is advisable to switch to a larger capacity syringe, or to change syringes to replenish NH_3_ (which can be done in under 2 min by a single operator). Although the amount of NH_3_ that can be supplied by the SPGD system has no advantage compared to some of previous studies (**Table 1**), we also recommend using multiple SPGD systems in parallel to simulate deposition in larger areas or at greater rates after weighing the cost. For the simulation of other compounds, e.g. NO_2_, SO_2_, their simulation methods have similarities with NH_3_, so the SPGD system may be equally useful, and multiple injectors in parallel can also be used for mixed deposition. When the air flow rate is constant, the SPGD system requires a higher-resolution syringe pump to adjust the NH_3_ concentration more accurately, which will increase the cost of components. Therefore, the resolution can also be improved by diluting the NH_3_ in the syringe by a certain multiple of air.

## 5 Conclusion

The proposed SPGD system is simple, stable and safe. A single SPGD system can simulate NH_3_/^15^NH_3_ deposition flux of 0 - 31.74 mg N m^-2^ d^-1^ (equivalent to 0 - 116 kg N ha^-1^ yr^-1^) over an area of 0.36 m^2^ using one 20 ml syringe. The system can be used for accurate NH_3_ deposition simulation or ^15^NH_3_ labeling of saplings or branches materials under greenhouse and indoor conditions. Overall, the SPGD system can provide a simpler, safer and lower-cost NH_3_/^15^NH_3_ deposition simulation solution for researchers working on gaseous deposition related issues.

## Data Availability

The original contributions presented in the study are included in the article/**Supplementary Material**. Further inquiries can be directed to the corresponding authors.
