# Supplementary material for: The Syringe Pump Gas Distribution (SPGD) system: a simple and low-cost method for simulating NH3/15NH3 deposition
Source: Front Plant Sci. 2025 Mar 28;16:1460035. doi: 10.3389/fpls.2025.1460035 (PMC12005220; doi:10.3389/fpls.2025.1460035)
Supplement: Supplementary file 1 [file DataSheet1.docx]

Supplementary Material

**Article title:**

The Syringe Pump Gas Distribution (SPGD) system: a simple and low-cost method for simulating NH_3_/^15^NH_3_ deposition

Authors: Chunze Wu, Xing Wei^*^, Chenghang Zhang, Saima Khan

The following Supporting Information is available for this article:

**Supplementary Table S1** Bill of components for a single SPGD system

**Supplementary Table S2** Parameter values to the calculation of the correction factor for NH_3_ adsorption on the chamber inner wall in this study

**Supplementary Figure S1.** A schematic diagram of the reaction apparatus

**Supplementary Figure S2.** Estimation of NH_3_ concentration in syringe by Water Absorption

**Supplementary Figure S3** Connection between air compressor, water separator and reduction valve in the laboratory

**Supplementary Figure S4** Connection between pneumatic quick connector, gas tube and activated charcoal filter in the laboratory.

**Supplementary Figure S5** Connection between Luer connection and gas mixer in the laboratory

**Supplementary Figure S6** Connection between reduction valve, activated charcoal filter and rotor flowmeter

**Supplementary Figure S7** Customized storage box and its internal components

**Supplementary Figure S8** Ambient temperature and relative humidity variations during SPGD system operation

**Supplementary Figure S9** NH_3_ concentrations at the inlet and outlet of the empty chamber

**Supplementary Figure S10** NH_3_ concentrations at the inlet and outlet of the chamber under LN and HN treatments.

**Supplementary Table S1** Bill of components for a single SPGD system.

| **Component name** | **Component** **description** | **No. units** |
| --- | --- | --- |
| Air compressor | 1580 watts, 230 L min^-1^ displacement (1580-30L-1, Taizhou Fujiwara Hardware Tools Co., Ltd.) | 1 |
| Water separator | Automatic drainage, 1/4” female threaded joints (AF2000, TWSNS®) | 1 |
| Reduction valve | 1/4” female threaded joints (AR2000, TWSNS®) | 1 |
| Activated charcoal filter | PMMA, 40 mm inner diameter, 280 mm long, 1/4” female threaded joints | 1 |
| Rotor flowmeter | 5-45 L min^-1^, 8 mm outer diameter pagoda joints (LZB-10, Changzhou Shuanghuan Thermal Instrument Co., Ltd.) | 1 |
| Microinjection pump | 3-channel, 0.01 mm min^-1^ resolution (QHZS-003A, Qhebot®) | 1 |
| Venturi gas mixer | PMMA, 40 mm outer diameter, 125 mm long, 1/2” male threaded joints | 1 |
| Pneumatic quick connector | Stainless steel, male,1/4” male threaded joints (PM-20, TWSNS®) | 2 |
|  | Stainless steel, female,1/4” male threaded joints (SM-20, TWSNS®) | 2 |
|  | Stainless steel, male, 8mm outer diameter pagoda joints (PH-20, TWSNS®) | 1 |
| Pagoda connector | Stainless steel, 8 mm outer diameter, 1/4” male threaded joints | 2 |
|  | Stainless steel, 6 mm outer diameter, 1/2” female threaded joints | 1 |
|  | Stainless steel, 8 mm outer diameter, 1/2” male threaded joints | 3 |
| Luer connector | PP, male Luer lock | 2 |
| Needle | Stainless steel and PP, 0.2 mm inner diameter, 0.4 mm outer diameter, female Luer lock | 1 |
| Gas tube | PTFE, 8 mm inner diameter, 10 mm outer diameter | 5 m |
|  | PTFE, 7 mm inner diameter, 9 mm outer diameter | 2 cm |
| Capillary tube | PTFE, 0.3 mm inner diameter, 0.6 mm outer diameter | 30 cm |
|  | PTFE, 2 mm inner diameter, 4 mm outer diameter | 5 cm |
| Syringe | PP, male Luer lock, 20 ml | 1 |
| Storage box | PP, 63 cm long, 42 cm wide, 33 cm high | 1 |
| Flowmeter bracket | PMMA, 116 mm center spacing | 1 |
| Corner bracket | Stainless steel, 120 mm long, 40 mm wide, 60 mm high | 2 |
| Rubber grommet | 14 mm inner diameter | 3 |
| Nuts and bolts | Stainless steel, Flange and Button, M6*12 mm | 4 |

The total cost is estimated at $249 per system, based on the actual cost from Chinese suppliers in 2023.

**Supplementary Table S2** Parameter values to the calculation of the correction factor for NH_3_ adsorption on the chamber inner wall in this study.

| **Parameters** | **Values** |
| --- | --- |
| ρ_In_ (μg N m^-3^) | 390.69 |
| ρ_Out(empty)_  (μg N m^-3^) | 343.07 |
| ρ_C_ (μg N m^-3^) | 47.62 |
| *S*_P_ (m^2^) | 0.36 |
| *S*_C_ (m^2^) | 2.19 |
| *S*_P_*/S*_C_ | 0.16 |
| ρ_X_ (μg N m^-3^) | 39.79 |

ρ_In_, the mean measured NH_3_ concentration at the inlet of the chamber; ρ_Out(empty)_, the mean measured NH_3_ concentration at the outlet of the chamber without plant-soil material; ρ_C_, the factor of NH_3_ adsorption on the inner wall of the chamber; *S*_P_, the vertical projection area of the plant-soil material; *S*_C_, the total area of the chamber inner wall; ρ_X_, the correction factor for NH_3_ adsorption on the chamber inner wall.

**Supplementary Figure S1.** A schematic diagram of the reaction apparatus. In the process of preparing NH_3_/^15^NH_3_ in laboratory, NH_4_Cl/^15^NH_4_Cl and Ca(OH)_2_ should be fully mixed outside the test tube and quickly loaded into the test tube to avoid waste of NH_3_/^15^NH_3_. Try to shorten the test tube or increase the amount of soda-lime to reduce the absorption of NH_3_ by the water generated by the reaction.


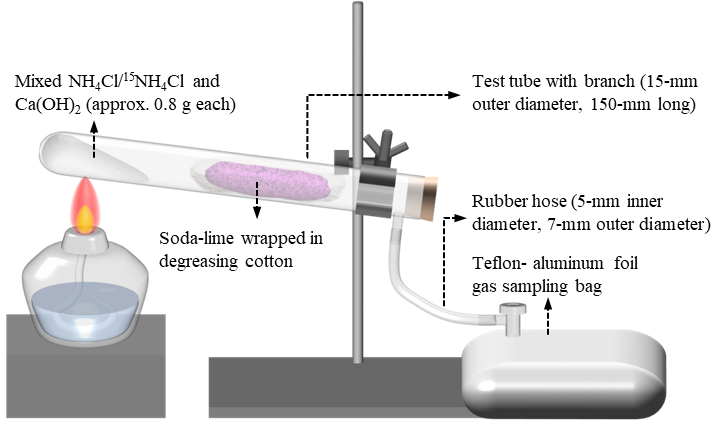


**Supplementary Figure S2.** Estimation of NH_3_ concentration in syringe by Water Absorption. When the prepared NH_3_ is nearly pure, the syringe piston will rebound in an instant. The NH_3_ concentration was estimated by the ratio of the remaining air volumes in the syringe before and after the water aspiration method (assuming that all NH_3_ will be absorbed by water).


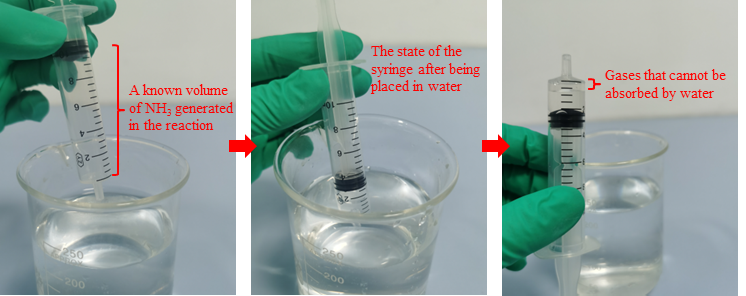


**Supplementary Figure S3** Connection between air compressor, water separator and reduction valve in the laboratory. Use pneumatic quick connectors to connect the air compressor, water separator and reduction valve.


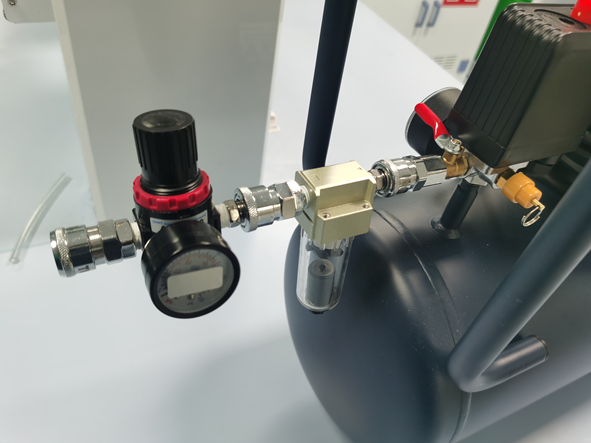


**Supplementary Figure S4** Connection between pneumatic quick connector, gas tube and activated charcoal filter in the laboratory. Use an 8 mm pagoda quick connector to connect the gas tube (40 cm), and use a pagoda connector to connect the other end of the tube to the activated carbon filter.


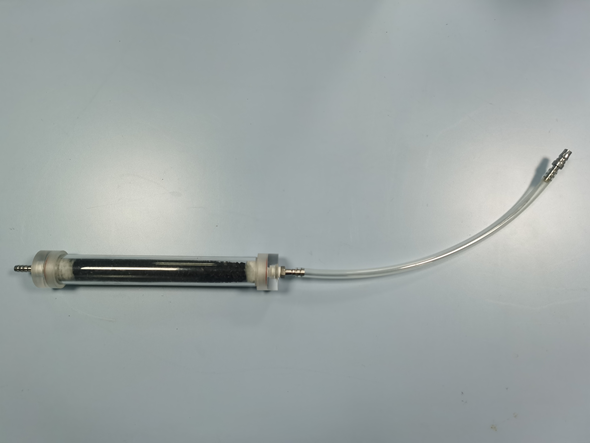


**Supplementary Figure S5** Connection between Luer connector and gas mixer in the laboratory. The purpose of using 2*4 mm PTFE tube is to make the NH_3_ concentration in the SPGD system stabilize faster.

**
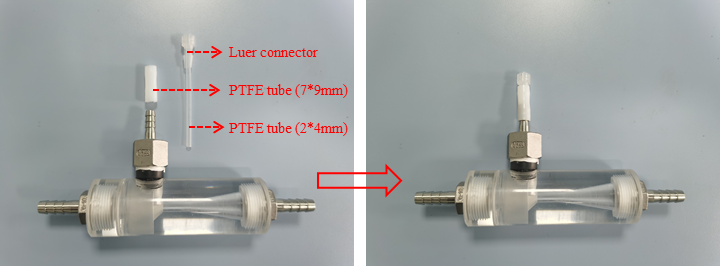
**

**Supplementary Figure S6** Connection between reduction valve, activated charcoal filter and rotor flowmeter.


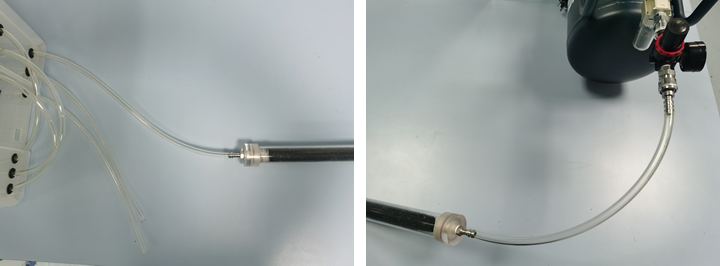


**Supplementary Figure S7** Customized storage box and its internal components. Drill 2 6-mm hole at the bottom of the storage box to install the flow meter bracket; drill 4 10-mm hole on the side to install the gas pipeline, and install a rubber ring around the hole to reduce wear on the gas pipeline; drill 2 6-mm hole on the side near the top to install the corner bracket for installing the control unit of the injection pump. We installed 3 SPGD systems in one packing box, so there are 8 6-mm and 12 10-mm holes.


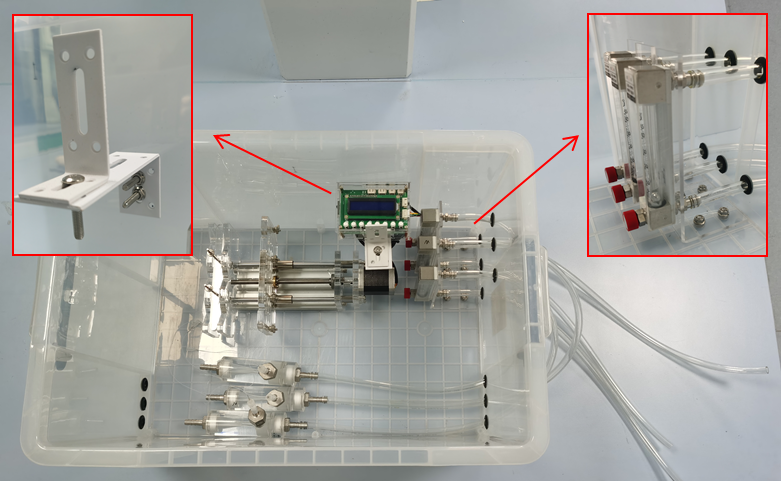


**Supplementary Figure S8** Ambient temperature and relative humidity variations during SPGD system operation.

**
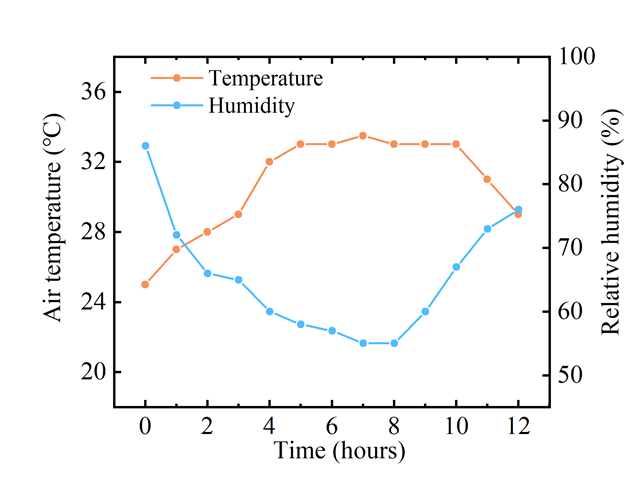
**

**Supplementary Figure S9** NH_3_ concentrations at the inlet and outlet of the empty chamber. Actual instrument readings were taken at 5-min intervals.

**
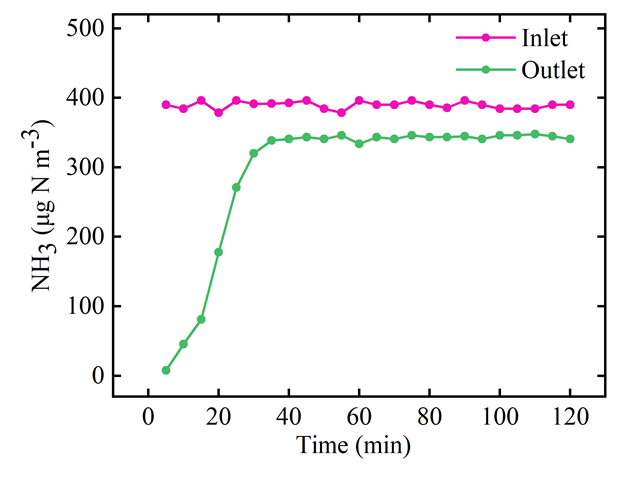
**

**Supplementary Figure S10** NH_3_ concentrations at the inlet and outlet of the chamber under LN (A) and HN (B) treatments. The black dotted lines indicate the theoretical value of NH_3_ concentration. Error bars indicate the standard error of the mean of NH_3_ concentration (*n* = 3).

**
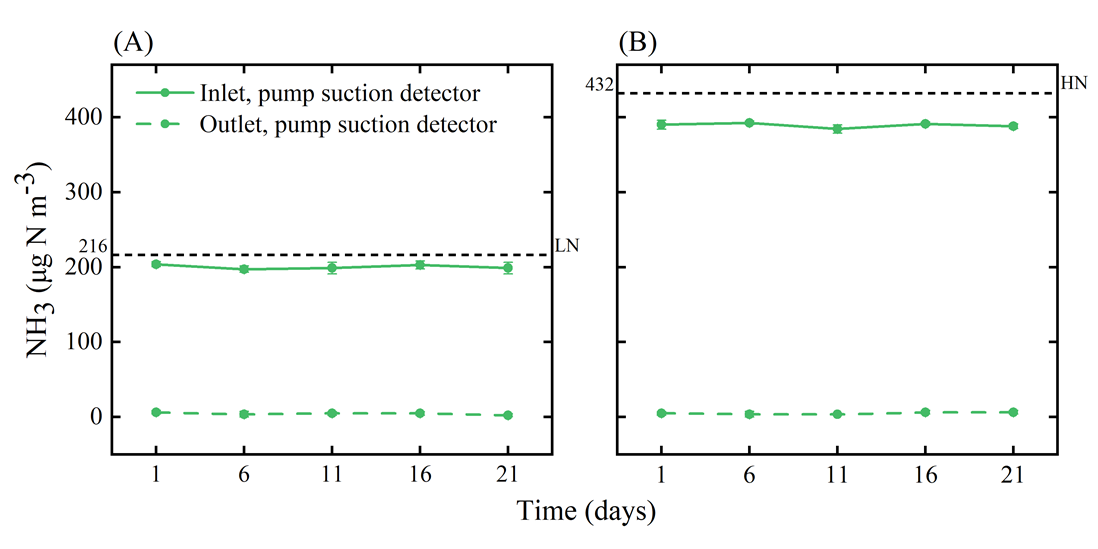
**
